# Structurally Diverse Small Molecule Quasiracemates

**DOI:** 10.1063/4.0000982

**Published:** 2025-10-27

**Authors:** Kraig Wheeler, Kamrynn Burk, Noah Dunham, Ainsley Hill, Molly Fleagle, Shay Perlot, Diana Schepens, Henry Zaske

**Affiliations:** Whitworth University, Spokane, WA, USA

## Abstract

Progress in the design of functional materials and supramolecular applications often emerges from a detailed understanding of molecular recognition events. This area continues to mature through the collective efforts of its practitioners by surveying the preferred orientations and conditional exceptions of molecular component and functional group associations. Our work in this area focuses on the quasiracemate approach for molecular assembly by examining the structural intricacies responsible for the pairwise construction of near enantiomers. Early attention to quasiracemates often embraced systems engineered using components with similar topological properties and only a single point of structural difference. This preferred method follows a pervasive belief, even today, that maximizing the success of small-molecule quasiracemate formation requires use of structurally close quasienantiomers. We demonstrate in this presentation that quasiracemate strategies do not require components with similar shape spaces and can even form with large imposed structural differences and multiple points of variance. These unprecedented quasiracemates are achieved using chiral benzoyl amino acids where the observed crystal motifs benefit from complementary molecular shapes and an extensive set of hydrogen bonds. Cocrystal assessment of these materials involved crystallographic, hot stage microscopy, and void space studies. Additionally, mathematical constructs were devised to help define the shape space variations of quasienantiomers and the degree of symmetry of the observed motifs.

